# Collismycin C from the Micronesian Marine Bacterium *Streptomyces* sp. MC025 Inhibits *Staphylococcus aureus* Biofilm Formation

**DOI:** 10.3390/md15120387

**Published:** 2017-12-12

**Authors:** Jin-Hyung Lee, Eonmi Kim, Hyukjae Choi, Jintae Lee

**Affiliations:** 1 School of Chemical Engineering, Yeungnam University, Gyeongsan-si 38541, Gyeongsangbukdo, Korea; jinhlee@ynu.ac.kr; 2 College of Pharmacy, Yeungnam University, Gyeongsan-si 38541, Gyeongsangbukdo, Korea; minnie60@hanmail.net

**Keywords:** collismycin C, antibiofilm activity, methicillin-resistant *Staphylococcus aureus*, iron chelation

## Abstract

Biofilm formation plays a critical role in antimicrobial resistance in *Staphylococcus aureus*. Here, we investigated the potential of crude extracts of 79 Micronesian marine microorganisms to inhibit *S. aureus* biofilm formation. An extract of *Streptomyces* sp. MC025 inhibited *S. aureus* biofilm formation. Bioactivity-guided isolation led to the isolation of a series of 2,2′-bipyridines: collismycin B (**1**), collismycin C (**2**), SF2738 D (**3**), SF2738 F (**4**), pyrisulfoxin A (**5**), and pyrisulfoxin B (**6**). Among these bipyridines, collismycin C (**2**) was found to be the most effective inhibitor of biofilm formation by methicillin-sensitive *S. aureus* and methicillin-resistant *S. aureus* (MRSA), and this compound inhibited MRSA biofilm formation by more than 90% at a concentration of 50 μg/mL. The antibiofilm activity of collismycin C was speculated to be related to iron acquisition and the presence and position of the hydroxyl group of 2,2′-bipyridines.

## 1. Introduction

The emerging rate of antibiotic resistance is a huge threat to public health [1]. In particular, *Staphylococcus aureus* is a major pathogen that frequently causes infections to the patients in the hospital and is well-known with high rate of antibiotic resistance such as methicillin-resistant *S. aureus* (MRSA) [2]. It is thus necessary to discover a new drug that can control the infection of *S. aureus* and MRSA.

It is known that *S. aureus* produces biofilms with extracellular polymeric substances and universally attaches to surface of organs and tissues. The polymeric biofilms function as a barrier to interfere the diffusion of antibiotics and protect pathogens against antibiotics [3,4]. Furthermore, subinhibitory concentrations of several antibiotics often increase biofilm formation [5,6,7]. Therefore, inhibition of biofilm formation of *S. aureus* is thought as a strategy to control infection of *S. aureus* without an additional increase in antibiotic resistance. 

Currently, there are several ways to inhibit microbial biofilm formation such as surface conditioning by surfactants [8], inhibiting production of adhesion molecules [9], suppressing biosynthesis of biofilm matrix [10], antagonizing microbial quorum sensing signaling [11], and killing microbes in biofilm [12].

In this study, the antibiofilm activities of the extracts of 79 cultured bacterial strains isolated from Micronesian marine organisms were evaluated, and a series of bipyridine compounds were purified from the bioactive extract of *Streptomyces* sp. MC025 (GenBank accession No. MG016024) by bioactivity-guided isolation. The antibiofilm activities of the isolated compounds were evaluated by biofilm formation assays in 96-well microtiter plates, and confocal microscopy, in order to identify biofilm inhibitors targeting *S. aureus* strains including methicillin-resistant *S. aureus* (MRSA). Furthermore, the antibiofilm mechanism of most active compound was studied. 

## 2. Results and Discussion 

As part of our research program to build a foundation for marine bioprospecting through the securement of overseas marine organisms, 79 bacterial strains were isolated from marine samples collected in the waters of Kosrae, Federated States of Micronesia in 2015. Based on 16S rDNA sequence analysis, the 79 strains were categorized into 30 different genera: three *Agrococcus* spp., one *Agromyces* sp., four *Alteromonas* spp., two *Aquimarina* spp., 14 *Bacillus* spp., one *Brachybacterium* sp., one *Ferrimonas* sp., two *Fictibacillus* spp., one *Jiangella* sp., three *Kocuria* spp., four *Loktanella* spp., two *Microbacterium* spp., one *Microbulbifer* sp., three *Micrococcus* spp., two *Nocardiopsis* spp., two *Paracoccus* spp., four *Photobacterium* spp., one *Planomicrobium* sp., four *Pseudoalteromonas* spp., one *Pseudomonas* sp., two *Pseudonocardia* sp., three *Pseudovibrio* spp., two *Rhodococcus* spp., one *Shewanella* sp., five *Staphylococcus* spp., four *Streptomyces* spp., five *Vibrio* spp., and one *Yangia* sp. (Table S1). EtOAc extracts of these 79 strains were initially screened for inhibition of *S. aureus* ATCC 6538 biofilm formation in 96-well microtiter plates at a concentration of 100 μg/mL. The bacterial extracts varied in their ability to control *S. aureus* biofilm formation; detailed information on *S. aureus* growth and biofilm formation is provided in Figure S1. Notably, three hits (MC009, MC025, and MC085) inhibited *S. aureus* biofilm formation by >78%. Based on 16S rRNA gene sequences, MC009 was identified as *Vibrio owensii* (GenBank accession No. MG16023), with a sequence similarity of 99.86% to *Vibrio owensii* DY05 (GenBank accession No. NR_117424), MC025 as *Streptomyces* sp. (GenBank accession No. MG016024), with a sequence similarity of 99.85% to *Streptomyces parvus* NBRC 14599 (GenBank accession No. NR_112437), and MC085 as *Aquimarina* sp. (GenBank accession No. MG016025), with 98.32% sequence similarity to *Aquimarina salinaria* antisso-27 (GenBank accession No. NR_108449). These three strains (MC009, MC025, and MC085) were isolated from an unidentified red tunicate, an unidentified red alga, and a consortium of unidentified marine tunicates, respectively.

**Figure 1:**
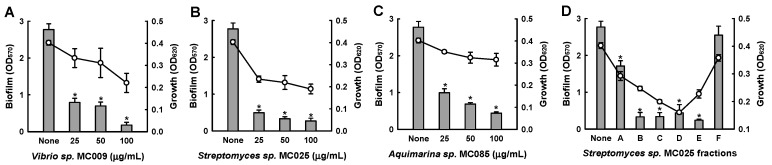
Inhibition of biofilm formation by extracts of marine microorganisms. Biofilm formation (OD_570_ as bars) and planktonic cell growth (OD_620_ as lines) by *S. aureus* ATCC 6538 were evaluated after 24 h of incubation in 96-well plates in the presence or absence of extracts of the marine microorganisms *Vibrio* sp. MC009 (**A**), *Streptomyces* sp. MC025 (**B**), and *Aquimarina* sp. MC085 (**C**), and fractions of the *Streptomyces* sp. MC025 extract (100 µg/mL) (**D**). *, *p* < 0.05 versus untreated controls.

Further biofilm formation assays showed that the extracts of MC009, MC025, and MC085 inhibited biofilm formation by *S. aureus* in a dose-dependent manner (Figure 1). In particular, at a concentration of 50 μg/mL, the extract of *Streptomyces* sp. MC025 decreased *S. aureus* ATCC 6538 biofilm formation by ≥90%, while planktonic cell growth slightly decreased. Among the three hits, the extract of *Streptomyces* sp. MC025 caused the greatest reduction in biofilm formation; additionally, the extract was subjected to NP vacuum liquid chromatography (VLC) to give six fractions, and four fractions (B, C, D, and E) markedly inhibited biofilm formation (Figure 1D). Hence, further chemical and biological investigation of the extract of *Streptomyces* sp. MC025 was performed. A plate image of *Streptomyces* sp. MC025 is provided in Figure S2.

**Figure 2:**
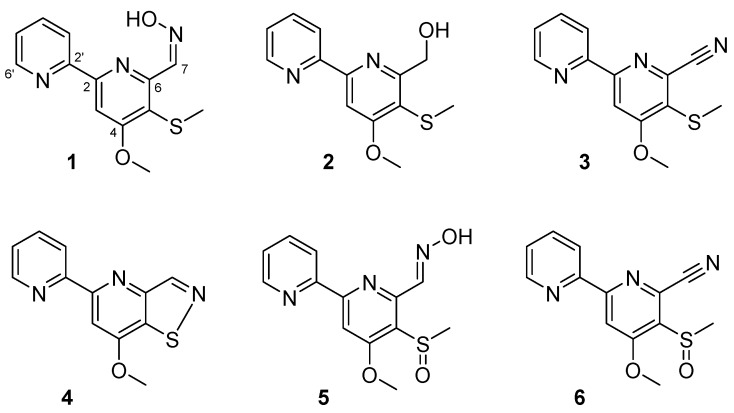
The structures of compounds **1**–**6**.

To identify the bioactive compounds responsible for inhibition of biofilm formation, a large-scale extract of *Streptomyces* sp. MC025 was prepared by EtOAc extraction of broth obtained from a 35 L culture of the strain. By bioactivity-guided fractionation on the crude extract, six compounds with 2,2′-bipyridine moieties were isolated: collismycins B and C (**1** and **2**), SF2738 D (**3**), SF2738 F (**4**), and pyrisulfoxins A and B (**5** and **6**) (Figure 2). 

Compound **1** was obtained as a white powder. The protonated molecule of **1** was observed at *m*/*z* 276.2 on HPLC-ESI-MS. The ^1^H and ^13^C NMR spectra of **1** (Figures S3 and S4, and Table S1) showed two distinct downfield-shifted methyl resonances (*δ*_H_/*δ*_C_ 4.16/56.9 and 2.42/18.5) corresponding to a methoxy group and a thiomethyl group, respectively. The four olefinic protons in a spin system [*δ*_H_ 8.71 (1H, ddd, H-6′, *J* = 4.8, 1.7, and 1.0 Hz), 8.12 (1H, ddd, H-3′, *J* = 8.0, 1.0, and 1.0 Hz), 7.87 (1H, ddd, H-4′, *J* = 8.0, 7.6, and 1.7 Hz) and 7.40 (1H, ddd, H-5′, *J* = 7.6, 4.8, and 1.0 Hz)] indicated the presence of a 2-substituted pyridine. The presence of an additional *sp*^2^ CH group [*δ*_H_ 8.13 (1H, s), *δ*_C_ 105.0] and four non-protonated carbons suggested the presence of a 2,4,5,6-substituted pyridine structure. The remaining resonances in the ^1^H and ^13^C NMR spectra [*δ*_H_ 8.70 (1H, d, H-7, *J* = 0.5 Hz), *δ*_C_ 140.4] showed chemical shifts typical of the aldoxime group. Based on MS and NMR spectroscopic data analysis, the structure of **1** was identified to be collismycin B [13].

Compound **2** was obtained as a white powder. The protonated molecule of **2** was observed at *m*/*z* 263.1 on LR-ESI-MS. The ^1^H and ^13^C NMR spectra of **2** (Figures S5 and S6) were similar to those of **1**. However, resonances corresponding to the aldoxime functional group in **1** were not observed, and ^1^H resonances [*δ*_H_ 4.93 (2H, s, H-7) and 4.78 (1H, br s, 7-OH)] corresponding to a hydroxymethyl group were newly observed. These observations, together with the comparison of the ^1^H and ^13^C NMR spectra of **2** with literature data, enabled the identification of **2** as collismycin C [13].

The protonated molecule of Compound **3** was observed at *m*/*z* 257.1 on HPLC-ESI-MS. The ^1^H and ^13^C NMR spectra of **3** (Figures S7 and S8) were similar to those of Compound **2**. However, the hydroxymethyl signals of **2** were not observed, and a non-protonated carbon peak (C-7) was observed at *δ*_C_ 116.6, indicating the presence of a nitrile group. Overall, the one-dimensional (1D) NMR spectra of **3** were found to be identical to those of a previously reported compound, SF2738 D [13].

Compounds **4**–**6** showed 1D NMR spectra almost identical to those of the other 2,2′-bipyridine compounds, and the protonated molecules for these compounds were observed at *m*/*z* 244.1, 292.1, and 274.1, respectively. Attempts at the dereplication of **4**–**6** gave several hits. After careful comparison of the experimental ^1^H and ^13^C NMR data (Figures S9–S14) with the data reported for these hit structures, we concluded that Compounds **4**–**6** are SF2738 F, pyrisulfoxin A, and pyrisulfoxin B, respectively [13,14].

**Figure 3:**
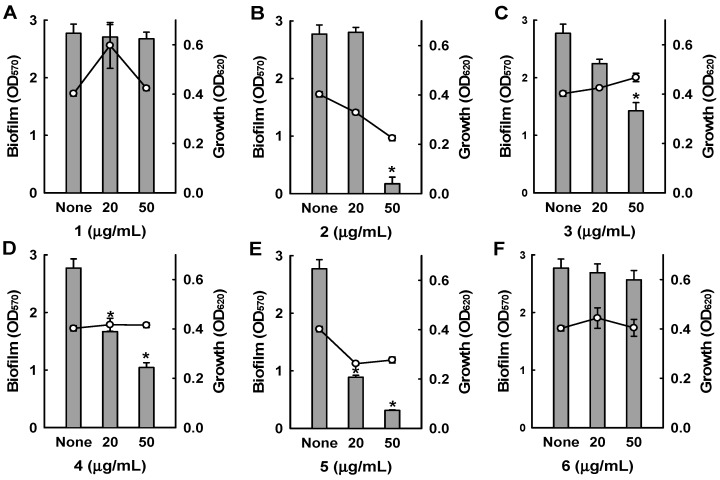
Inhibition of biofilm formation by **1**–**6**. Biofilm formation (OD_570_ as bars) and planktonic cell growth (OD_620_ as lines) by *S. aureus* 6538 were quantified after 24 h incubation in 96-well plates in the presence or absence of the isolated compounds. * *p* < 0.05 versus untreated controls.

**Figure 4:**
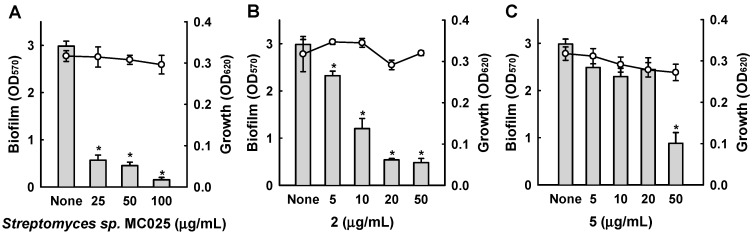
Antibiofilm activities of collismycins against a methicillin-resistant *S. aureus* strain (MRSA). The antibiofilm activities of *Streptomyces* sp. MC025 extract, collismycin C (**2**), and pyrisulfoxin A (**5**) were quantified by measuring biofilm formation (OD_570_ as bars) and planktonic cell growth (OD_620_ as lines) by a methicillin-resistant *S. aureus* strain (MRSA, ATCC 33591) in the presence or absence of these components. * *p* < 0.05 versus untreated controls.

Among the six bipyridines isolated (**1**–**6**), Compounds **2**–**5** showed antibiofilm activity against methicillin-sensitive *S. aureus* ATCC 6538 at 50 µg/mL (Figure 3). Compounds **2** and **5** were two of the major products of bioactivity-guided isolation, and these compounds showed more potent antibiofilm activity than the other isolates at a concentration of 50 μg/mL. We also examined the effects of the extract of *Streptomyces* sp. MC025, **2**, and **5** on methicillin-resistant *S. aureus* (MRSA) biofilm formation and cell growth at concentrations ranging from 5 to 50 μg/mL. As expected, the extract of *Streptomyces* sp. MC025 and collismycin C (**2**) both inhibited *S. aureus* biofilm formation in a dose-dependent manner without affecting the growth of planktonic cells (Figure 4A,B), while pyrisulfoxin A (**5**) was less active against MRSA than against methicillin-sensitive *S. aureus* ATCC 6538 (Figure 4C). These results suggest that collismycin C is a major component of the *Streptomyces* sp. MC025 extract with antibiofilm activity against *S. aureus* ATCC 6538 and MRSA. It is speculated that the presence and position of the hydroxyl group on these bipyridines are critical for antibiofilm activity against *S. aureus*.

**Figure 5:**
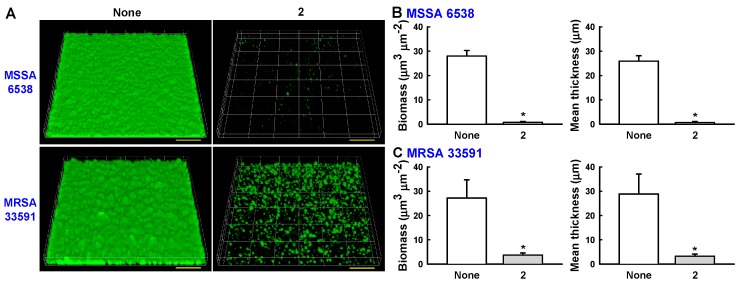
(**A**) Observation of biofilm formation by confocal laser microscopy. Scale bars represent 100 μm. (**B**) Quantification of biofilm formation of MSSA 6538 using COMSTAT software. (**C**) Quantification of biofilm formation of MRSA 33591 using COMSTAT software.

Confocal laser microscopy was also used to analyze changes in biofilm formation. In line with the quantitative data from the biofilm formation assays in 96-well plates (Figure 3 and Figure 4), fluorescence images indicated that collismycin C at 50 µg/mL markedly inhibited biofilm formation by two *S. aureus* strains (Figure 5A). Inhibition of biofilm formation was confirmed by measuring biofilm quantity in COMSTAT software. Collismycin C reduced the biomass (volume/area) and mean thickness of *S. aureus* 6538 biofilms by >98% (Figure 5B) and reduced the biomass of MRSA biofilms by 90% (Figure 5C).

Several natural products containing 2,2′-bipyridine structures, including caerulomycins [15,16,17,18], SF2738 A-F [13], collismycins [19], and pyrisulfoxins [14], have been reported to have antimicrobial, cytotoxic, and anti-inflammatory activities; these compounds have been isolated from *Streptomyces caeruleus*, *Streptomyces* sp. SF2738, *Streptomyces* sp. MQ22, and *Streptomyces californicus*. Caerulomycin A, possessing 4-*O*-methyl and 6-*E*-aldoxime groups, is known to act as an antibiotic [15], anti-asthma agent [20], and immunosuppressive agent [21]. Caerulomycin C, which has 3,4-di-*O*-methyl and 6-*E*-aldoxime groups, showed similar antibiotic activity [16]. Fu et al. speculated that the antimicrobial properties of caerulomycins result from their oxime functionalities [18]. Pyrisulfoxin A, which has 4-*O*-methyl and 6-*E*-aldoxime groups, exhibits cytotoxicity against P388 murine leukemia cells [14]. SF2738 A (also reported as collismycin B, **1**) and SF2738 B (collismycin A), which both have 4-*O*-methyl, 5-*S*-methyl, and 6-aldoxime groups, have been revealed to possess weak antibacterial activities (but no activity against *Staphylococcus aureus* Smith S-424 or *S. aureus* 209P), broad but weak antifungal activities, and cytotoxicity against P388 leukemia cells, with IC_50_ values of 0.08 and 0.25 μg/mL, respectively [13]. Collismycin C (**2**) has been reported to possess poor antimicrobial and cytotoxic activities, and to have weaker antimicrobial and cytotoxic activities than collismycin B (**1**) [13]. However, in our screen for antibiofilm activity against *S. aureus*, **2** showed more potent activity than the other active isolates (**3**–**5**) despite lacking an aldoxime functional group, while **1**, which has an aldoxime group, showed no antibiofilm activity at 50 μg/mL. These observations indicate that the antibiofilm activities of collismycins do not directly correspond to their antibacterial activities, and that these two activities of collismycins might be achieved by different mechanisms or different combinations of mechanisms. 

**Figure 6:**
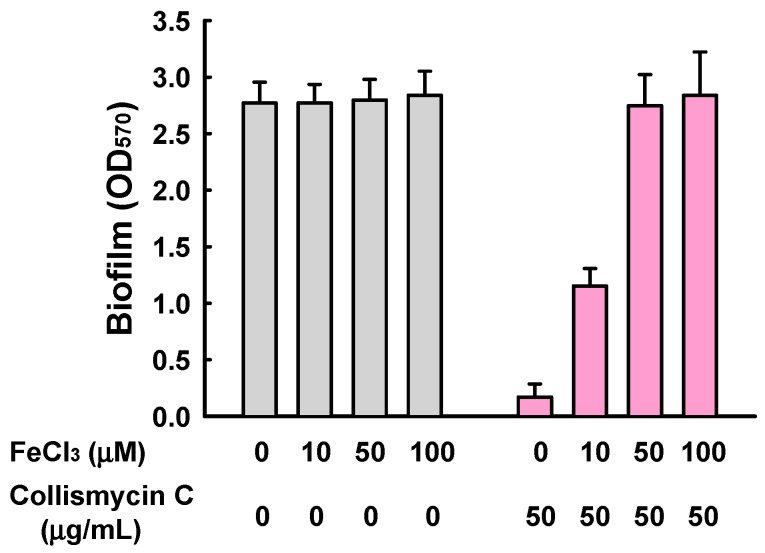
Effect of the addition of exogenous iron on *S. aureus* biofilm formation in the presence of collismycin C (**2**). Biofilm formation (OD_570_) by *S. aureus* ATCC 6538 was quantified after 24 h incubation in 96-well plates in the presence or absence of FeCl_3_ with (pink) or without (grey) the treatment of collismycin C.

2,2′-Bipyridine-containing compounds have also been extensively investigated as metal ion chelators [22], and iron ions are accepted as being essential for biofilm formation by diverse microbes, including *Pseudomonas aeruginosa* [23] and *Staphylococcus aureus* [24]. More recently, collismycin A has been revealed to inhibit cancer cell growth by chelating Fe^2+^ and Fe^3+^ ions [25]. In addition, while SF2738 D (**3**) and SF2738 F (**4**) showed mild antibiofilm activities in this study, these compounds displayed no antibacterial, antifungal, or cytotoxic activities in previous screening experiments [13]. We therefore investigated the effect of exogenous iron addition on *S. aureus* biofilm formation in the presence of **2**. The addition of FeCl_3_ together with **2** clearly restored *S. aureus* biofilm formation in a dose-dependent manner, while the addition of FeCl_3_ alone did not significantly affect biofilm formation (Figure 6). Therefore, collismycin C, like collismycin A, acts as an iron chelator, and the antibiofilm activities of collismycins can be speculated to be the result of iron chelation in iron-limited media. However, despite the presence of 2,2′-bipyridine moieties, **1** and **6** did not inhibit *S. aureus* biofilm formation, indicating that multiple factors might affect the antibiofilm activities of 2,2′-bipyridines, including the type and position of their substituents.

In this study, collismycin C (**2**) was identified as a potent antibiofilm agent, which inhibits biofilm formation by both MSSA and MRSA by chelating iron ions. Collismycin C was previously reported with very weak cytotoxicity against P388 murine leukemia cells (IC_50_ = 28.6 μM) with poor antibacterial activities [13]. Therefore, collismycin C could be used as a lead to develop anti-infective agents with antibiofilm properties against MSSA and MRSA.

## 3. Materials and Methods 

### 3.1. General Experimental Procedures

^1^H and ^13^C NMR spectra were obtained using a Bruker Avance DPX-250 spectrometer. NMR experiments were performed at 294 K, using CDCl_3_ as a solvent. Coupling constants (*J*) were measured in Hz. LR-ESI-MS spectra were recorded using an Agilent Technologies 6120 quadrupole LC/MS system with a C18 column (Phenomenex Luna 3μ C18(2) M, 100 Å, New column; 150 × 4.6 mm) at a flow rate of 0.7 mL/min. HPLC was performed using a WATERS 1525 binary HPLC pump equipped with a WATERS 996 photodiode array detector together with a Hector C18 (250 × 21.2 mm) reversed-phase HPLC column.

### 3.2. Isolation of Microbial Strains from Micronesian Marine Samples

A red alga specimen (15C070) was collected by SCUBA in Kosrae, Micronesia in 2015 and cut into small pieces. A piece of red alga was squeezed to prepare sap, and 1 μL of sap was diluted with 1.0 mL of filtered and sterilized seawater. The resulting mixture was spread onto SYP SW agar (soluble starch, 10 g; yeast extract, 4 g; peptone, 2 g; Bacto agar, 15 g; filtered seawater, 1 L) and incubated at room temperature. After incubation for one week, a colony was picked from the crude plate and transferred onto a fresh SYP SW agar plate. An axenic culture of the bacterial strain MC025 was produced by repeated inoculation. Based on 16S rDNA sequence analysis, this strain was identified as *Streptomyces* sp., with 99.85% similarity to *Streptomyces parvus* NBRC 14599. An additional 98 bacterial strains were isolated from the biomass collected by SCUBA using the same protocol.

### 3.3. Small-Scale Fermentation and Extraction

To screen the isolated bacterial strains for antibiofilm activity, each bacterial strain was inoculated into SYP SW liquid medium (2 L) and incubated for one week (25 °C with shaking at 150 rpm). The cultured broth was extracted twice with EtOAc and dried under a stream of N_2_ gas. The bioactive crude extract of *Streptomyces* sp. MC025 was separated by normal-phase (NP) silica gel column chromatography using step-gradient elution with a solvent mixture of CH_2_Cl_2_ and MeOH. Six fractions (Fr. A–F) were collected, of which fractions B–E showed inhibitory effects on biofilm formation.

### 3.4. Isolation of **1**–**6** from Large-Scale Culture Broth of Streptomyces sp. MC025

*Streptomyces* sp. MC025 was cultured in SYP SW liquid medium (35 × 1 L) for 7 days at 25 °C with shaking at 150 rpm, and the resulting broth was extracted twice with EtOAc. The combined extract was evaporated under reduced pressure to yield 2.3 g of crude material. The extract was separated into six fractions (Fr. A–F) by NP VLC (silica gel) using step-gradient elution with a solvent mixture of CH_2_Cl_2_ and MeOH. These fractions were analyzed by LC/MS for comparison with the corresponding fractions from small-scale cultures. Fractions D and E were subjected to reversed-phase HPLC (Hector C18; 250 × 21.2 mm; 6 mL/min) with an acetonitrile–H_2_O gradient from 48:52 to 58:42 (*v*/*v*). Under these conditions, collismycin B (**1**, 8.0 mg), collismycin C (**2**, 18.8 mg), SF2738 D (**3**, 4.3 mg), SF2738 F (**4**, 6.6 mg), and pyrisulfoxin B (**6**, 6.2 mg) were purified with retention times of 17, 30, 35, 50, and 47 min, respectively. Fraction F was subjected to reversed-phase HPLC (25% acetonitrile in H_2_O; 6 mL/min) to give pure pyrisulfoxin A (**5**, RT 25 min; 40.7 mg).

Collismycin B (**1**): white powder; ^1^H NMR (CDCl_3_, 250 MHz) and ^13^C NMR (CDCl_3_, 63 MHz) data, see supplementary materials (Table S1, Figures S3 and S4); LR-ESIMS *m*/*z* 276.2 [M + H]^+^ (calcd. for C_13_H_14_N_3_O_2_S, 276.1).

Collismycin C (**2**): white powder; ^1^H NMR (CDCl_3_, 250 MHz) and ^13^C NMR (CDCl_3_, 63 MHz) data, see supplementary materials (Table S1, Figures S5 and S6); LR-ESIMS *m*/*z* 263.1 [M + H]^+^ (calcd. for C_13_H_15_N_2_O_2_S, 263.1).

SF2738 D (**3**): white powder; ^1^H NMR (CDCl_3_, 250 MHz) and ^13^C NMR (CDCl_3_, 63 MHz) data, see supplementary materials (Table S1, Figures S7 and S8); LR-ESIMS *m*/*z* 257.1 [M + H]^+^ (calcd. for C_13_H_12_N_3_OS, 258.1).

SF2738 F (**4**): white powder; ^1^H NMR (CDCl_3_, 250 MHz) and ^13^C NMR (CDCl_3_, 63 MHz) data, see supplementary materials (Table S1, Figures S9 and S10); LR-ESIMS *m*/*z* 244.1 [M + H]^+^ (calcd. for C_12_H_10_N_3_OS, 244.1).

Pyrisulfoxin A (**5**): white powder; ^1^H NMR (CDCl_3_, 250 MHz) and ^13^C NMR (CDCl_3_, 63 MHz) data, see supplementary materials (Table S1, Figures S11 and S12); LR-ESIMS *m*/*z* 292.1 [M + H]^+^ (calcd. for C_13_H_14_N_3_O_3_S, 292.1).

Pyrisulfoxin B (**6**): white powder; ^1^H NMR (CDCl_3_, 250 MHz) and ^13^C NMR (CDCl_3_, 63 MHz) data, see supplementary materials (Table S1, Figures S13 and S14); LR-ESIMS *m*/*z* 274.1 [M + H]^+^ (calcd. for C_13_H_12_N_3_O_2_S, 274.1).

### 3.5. Biofilm-Forming Bacterial Strains and Culture Conditions

*S. aureus* (ATCC 6538) and MRSA (ATCC 33591) were used in this study. All experiments were conducted in Luria-Bertani (LB) medium at 37 °C. Bacteria were initially streaked from −80 °C glycerol stocks onto LB plates, and a fresh single colony was inoculated into 25 mL LB medium in a 250 mL flask and, shaken at 250 rpm, cultured overnight at 37 °C. Overnight cultures were re-inoculated into LB medium at a dilution of 1:100. Cell growth in the presence of different concentrations of compounds was monitored by measuring absorbance at 620 nm (OD_620_) using a spectrophotometer (UV-160, Shimadzu, Japan). All experiments were performed using at least two independent cultures.

### 3.6. Antibiofilm Assays

A static biofilm formation assay was performed in 96-well polystyrene plates (SPL Life Sciences, Pocheon, Korea), as previously described. [26] Briefly, cells were inoculated into LB medium (for MSSA ATCC 6538) or LB supplemented with 0.2% glucose (for MRSA ATCC 33591), at an initial OD_600_ of 0.05 in a total volume of 300 µL. The cells were then cultured with or without the test compounds for 24 h without shaking. Biofilms in 96-well plates were stained with crystal violet and dissolved in 95% ethanol, and absorbance at 570 nm (OD_570_) was measured to quantify total biofilm formation. Cell growth in 96-well plates was also monitored by measuring absorbance at 620 nm (OD_620_). Results represent the mean of at least 12 replicate wells.

### 3.7. Confocal Laser Microscopy

Static biofilms in 96-well plates were visualized by confocal laser microscopy (Nikon Eclipse Ti, Nikon Instruments, Tokyo, Japan) using an Ar laser (excitation 488 nm, emission 500–550 nm) and a 20× objective. Color confocal images were produced using NIS-Elements C version 3.2 (Nikon Instruments, Tokyo, Japan). For each experiment, at least 10 random positions in each of three independent cultures were chosen for microscopic analysis. To quantify biofilm formation in the presence and absence of collismycin C, COMSTAT biofilm software (kindly provided by Arne Heydorn, Søborg, Denmark) was used to determine biomass (μm^3^/μm^2^) and mean thickness (μm). At least four positions and 20 planar images per position were analyzed.

## Supplementary Materials

The following are available online at www.mdpi.com/1660-3397/15/12/387/S1. Figure S1: Antibiofilm activity screening of the extracts of 79 bacterial culture broth; Figure S2: Plate image of *Streptomyces* sp. MC025; Figures S3–S14: ^1^H and ^13^C NMR spectra (250 and 63 MHz, respectively; CDCl_3_) of **1**–**6**; Table S1: List of 79 bacterial strains isolated from marine organisms collected in the waters of Kosrae, Federated States of Micronesia; Table S2: ^1^H and ^13^C NMR chemical shift data (250 and 63 MHz, respectively; CDCl_3_) of **1**–**6**.
