# Supplementary material for: Collismycin C from the Micronesian Marine Bacterium Streptomyces sp. MC025 Inhibits Staphylococcus aureus Biofilm Formation
Source: Mar Drugs. 2017 Dec 12;15(12):387. doi: 10.3390/md15120387 (PMC5742847; doi:10.3390/md15120387)
Supplement: Supplementary file 1 [file marinedrugs-15-00387-s001.pdf]

Supplementary Materials

Collismycin C from the Micronesian Marine Bacterium *Streptomyces* sp. MC025 Inhibits *Staphylococcus aureus* Biofilm Formation

Jin-Hyung Lee [1,†], Eonmi Kim [2,†], Hyukjae Choi [2,*] and Jintae Lee [1,*]

[1] School of Chemical Engineering, Yeungnam University, Gyeongsan-si, Gyeongsangbukdo 38541, Korea; jinhlee@ynu.ac.kr (J.H.L.)

[2] College of Pharmacy, Yeungnam University, Gyeongsan-si, Gyeongsangbukdo 38541, Korea; minnie60@hanmail.net (E.K.)

* Correspondence: h5choi@yu.ac.kr (H.C.); jtlee@ynu.ac.kr (J. L.); Tel.: +82-53-810-2824 (H.C.); +82-53-810-2533

† These authors equally contributed to this work.

Table of Contents

		Page
**Table S1.**	List of 79 bacterial strains isolated from marine organisms collected in the waters of Kosrae, Federated States of Micronesia	3 – 5
**Table S2.**	^{1}H and ^{13}C NMR (250 and 63 MHz, respectively; $CDCl_3$) data of **1-6**	6
**Figure S1.**	Antibiofilm activity screening of the extracts of 79 bacterial culture broth	7
**Figure S2.**	Plate image of *Streptomyces* sp. MC025	8
**Figure S3.**	^{1}H NMR spectrum of compound **1** ($CDCl_3$, 250 MHz)	9
**Figure S4.**	^{13}C NMR spectrum of compound **1** ($CDCl_3$, 63 MHz)	10
**Figure S5.**	^{1}H NMR spectrum of compound **2** ($CDCl_3$, 250 MHz)	11
**Figure S6.**	^{13}C NMR spectrum of compound **2** ($CDCl_3$, 63 MHz)	12
**Figure S7.**	^{1}H NMR spectrum of compound **3** ($CDCl_3$, 250 MHz)	13
**Figure S8.**	^{13}C NMR spectrum of compound **3** ($CDCl_3$, 63 MHz)	14
**Figure S9.**	^{1}H NMR spectrum of compound **4** ($CDCl_3$, 250 MHz)	15
**Figure S10.**	^{13}C NMR spectrum of compound **4** ($CDCl_3$, 63 MHz)	16
**Figure S11.**	^{1}H NMR spectrum of compound **5** ($CDCl_3$, 250 MHz)	17
**Figure S12.**	^{13}C NMR spectrum of compound **5** ($CDCl_3$, 63 MHz)	18
**Figure S13.**	^{1}H NMR spectrum of compound **6** ($CDCl_3$, 250 MHz)	19
**Figure S14.**	^{13}C NMR spectrum of compound **6** ($CDCl_3$, 63 MHz)	20

Table S1. List of 79 bacterial strains isolated from marine organisms collected in the waters of Kosrae, Federated States of Micronesia

Strain	Phylum	Class	Blast Top	Similarity	sequence length
MC001	Proteobacteria	γ-Proteobacteria	Photobacterium ganghwense strain FR1311	99.43780745	1415/1423
MC006	Proteobacteria	γ-Proteobacteria	Alteromonas macleodii str. 'Balearic Sea AD45'	99.85041137	1335/1337
MC007	Firmicutes	Bacilli	Bacillus aryabhattai strain B8W22	99.92952784	1418/1419
MC008	Proteobacteria	γ-Proteobacteria	Vibrio maritimus strain R-40493	98.64864865	1387/1406
MC009	Proteobacteria	γ-Proteobacteria	Vibrio owensii strain DY05	99.85559567	1383/1385
MC010	Actinobacteria	Actinobacteria	Nocardioides furvisabuli strain SBS-26	99.40784604	1343/1351
MC011	Actinobacteria	Actinobacteria	Rhodococcus ruber strain DSM 43338	99.06069364	1371/1384
MC012	Bacteroidetes	Flavobacteria	Aquimarina spongiae strain A6	98.93162393	1389/1404
MC015	Firmicutes	Bacilli	Staphylococcus saprophyticus strain ATCC 15305	99.92810927	1390/1391
MC016	Proteobacteria	α-Proteobacteria	Pseudovibrio sp. FO-BEG1 strain FO-BEG1	98.44329133	1328/1349
MC020	Actinobacteria	Actinobacteria	Kocuria marina strain KMM 3905	99.71305595	1390/1394
MC022	Proteobacteria	α-Proteobacteria	Loktanella soesokkakensis strain DSSK1-5	99.92779783	1384/1385
MC023	Proteobacteria	γ-Proteobacteria	Alteromonas macleodii str. 'Balearic Sea AD45'	99.85663082	1393/1395
MC024	Actinobacteria	Actinobacteria	Micrococcus yunnanensis strain YIM 65004	99.70760234	1364/1368
MC025	Actinobacteria	Actinobacteria	Streptomyces parvus strain NBRC 14599	99.8540146	1368/1370
MC026	Actinobacteria	Actinobacteria	Brachybacterium paraconglomeratum strain LMG 19861	100	1382/1382
MC028	Proteobacteria	γ-Proteobacteria	Ferrimonas futtsuensis strain FUT3661	99.12600146	1361/1373
MC030	Firmicutes	Bacilli	Staphylococcus arlettae strain ATCC 43957	100	1392/1392
MC031	Firmicutes	Bacilli	Bacillus megaterium strain NBRC 15308	100	1416/1416
MC033	Firmicutes	Bacilli	Bacillus toyonensis strain BCT-7112	99.85925405	1419/1421
MC034	Proteobacteria	α-Proteobacteria	Loktanella pyoseonensis strain JJM85	99.16981132	1314/1325
MC035	Proteobacteria	γ-Proteobacteria	Pseudomonas brenneri strain CFML 97-391	99.56865564	1385/1391
MC036	Firmicutes	Bacilli	Planomicrobium okeanokoites strain NBRC 12536	99.85347985	1363/1365
MC037	Proteobacteria	γ-Proteobacteria	Alteromonas macleodii strain NBRC 102226	98.5	1379/1400
MC038	Firmicutes	Bacilli	Bacillus aryabhattai strain B8W22	100	1416/1416
MC041	Actinobacteria	Actinobacteria	Agrococcus terreus strain DNG5	99.70887918	1370/1374
MC042	Firmicutes	Bacilli	Fictibacillus phosphorivorans strain Ca7	100	1340/1340
MC047	Actinobacteria	Actinobacteria	Streptomyces albolongus strain NBRC 13465	99.27588704	1371/1381
MC051	Actinobacteria	Actinobacteria	Pseudonocardia kongjuensis strain LM 157	98.85386819	1380/1396
MC053	Actinobacteria	Actinobacteria	Pseudonocardia antitumoralis strain SCSIO 01299	99.78601997	1399/1402

MC054	Proteobacteria	γ-Proteobacteria	Vibrio furnissii strain 9119-82	99.71448965	1397/1401
MC055	Proteobacteria	γ-Proteobacteria	Microbulbifer variabilis strain Ni-2088	99.12727273	1363/1375
MC056	Proteobacteria	γ-Proteobacteria	Pseudoalteromonas shioyasakiensis strain SE3	99.2074928	1377/1388
MC058	Proteobacteria	γ-Proteobacteria	Photobacterium ganghwense strain FR1311	99.20692141	1376/1387
MC061	Actinobacteria	Actinobacteria	Agrococcus terreus strain DNG5	99.49640288	1383/1390
MC062	Actinobacteria	Actinobacteria	Agrococcus terreus strain DNG5	99.78229318	1375/1378
MC063	Actinobacteria	Actinobacteria	Microbacterium neimengense strain 7087	98.61516035	1353/1372
MC066	Actinobacteria	Actinobacteria	Microbacterium aquimaris strain JS54-2	99.19531822	1356/1367
MC067	Firmicutes	Bacilli	Staphylococcus gallinarum strain VIII1	100	1307/1307
MC068	Actinobacteria	Actinobacteria	Micrococcus yunnanensis strain YIM 65004	99.9271137	1371/1372
MC070	Firmicutes	Bacilli	Staphylococcus capitis strain JCM 2420	99.85611511	1388/1390
MC072	Proteobacteria	γ-Proteobacteria	Photobacterium rosenbergii strain CC1	99.57050823	1391/1397
MC073	Proteobacteria	α-Proteobacteria	Paracoccus zeaxanthinifaciens strain R-1506	99.78479197	1391/1394
MC074	Firmicutes	Bacilli	Bacillus cibi strain JG-30	99.92790195	1386/1387
MC075	Proteobacteria	γ-Proteobacteria	Pseudoalteromonas shioyasakiensis strain SE3	99.56521739	1374/1380
MC076	Proteobacteria	α-Proteobacteria	Yangia pacifica strain DX5-10	98.70722433	1298/1315
MC077	Proteobacteria	α-Proteobacteria	Pseudovibrio sp. FO-BEG1 strain FO-BEG1	99.92581602	1347/1348
MC078	Proteobacteria	γ-Proteobacteria	Pseudoalteromonas shioyasakiensis strain SE3	99.42028986	1372/1380
MC081	Actinobacteria	Actinobacteria	Streptomyces globisporus strain KCTC 9026	100	1312/1312
MC082	Firmicutes	Bacilli	Bacillus cereus ATCC 14579	100	1403/1403
MC085	Bacteroidetes	Flavobacteria	Aquimarina salinaria strain antisso-27	98.31994156	1346/1369
MC086	Proteobacteria	α-Proteobacteria	Loktanella soesokkakensis strain DSSK1-5	99.8470948	1306/1308
MC090	Proteobacteria	γ-Proteobacteria	Photobacterium ganghwense strain FR1311	99.64739069	1413/1418
MC091	Proteobacteria	α-Proteobacteria	Loktanella soesokkakensis strain DSSK1-5	99.92779783	1384/1385
MC093	Firmicutes	Bacilli	Bacillus infantis	99.71771348	1413/1417
MC094	Proteobacteria	γ-Proteobacteria	Shewanella haliotis strain DW01	99.85663082	1393/1395
MC097	Firmicutes	Bacilli	Bacillus aquimaris strain TF-12	98.9154013	1368/1383
MC099	Firmicutes	Bacilli	Staphylococcus arlettae strain ATCC 43957	99.85326486	1361/1363
MC100	Proteobacteria	γ-Proteobacteria	Vibrio alginolyticus strain NBRC 15630	99.85559567	1383/1385
MC101	Actinobacteria	Actinobacteria	Nocardiopsis prasina strain DSM 43845	99.77186312	1312/1315
MC102	Proteobacteria	γ-Proteobacteria	Vibrio alginolyticus strain NBRC 15630	99.92857143	1399/1400
MC103	Actinobacteria	Actinobacteria	Jiangella muralis strain 15-Je-017	98.34462002	1307/1329

MC104	Firmicutes	Bacilli	Bacillus endophyticus strain 2DT	99.55156951	1332/1338
MC106	Actinobacteria	Actinobacteria	Streptomyces coelicoflavus strain NBRC 15399	100	1378/1378
MC107	Firmicutes	Bacilli	Bacillus infantis	99.85724483	1399/1401
MC109	Firmicutes	Bacilli	Bacillus cereus ATCC 14579	99.85905567	1417/1419
MC111	Proteobacteria	γ-Proteobacteria	Alteromonas macleodii str. 'Balearic Sea AD45'	99.92679356	1365/1366
MC112	Firmicutes	Bacilli	Bacillus oceanisediminis strain H2	99.63924964	1381/1386
MC115	Firmicutes	Bacilli	Fictibacillus barbaricus strain V2-BIII-A2	99.3598862	1397/1406
MC116	Proteobacteria	α-Proteobacteria	Paracoccus zeaxanthinifaciens strain R-1506	99.85590778	1386/1388
MC117	Proteobacteria	γ-Proteobacteria	Pseudovibrio sp. FO-BEG1 strain FO-BEG1	100	1342/1342
MC119	Actinobacteria	Actinobacteria	Kocuria palustris strain TAGA27	100	1370/1370
MC120	Actinobacteria	Actinobacteria	Agromyces indicus strain NIO-1018	99.77810651	1349/1352
MC121	Actinobacteria	Actinobacteria	Micrococcus sp. MOLA 4	99.93127148	1454/1455
MC122	Proteobacteria	γ-Proteobacteria	Pseudoalteromonas shioyasakiensis strain SE3	99.70695971	1361/1365
MC125	Firmicutes	Bacilli	Bacillus subtilis subsp. spizizenii strain ATCC 6633	100	1400/1400
MC127	Firmicutes	Bacilli	Bacillus aerius strain 24K	100	1400/1400
MC129	Actinobacteria	Actinobacteria	Rhodococcus equi strain DSM 20307	100	1360/1360
MC130	Actinobacteria	Actinobacteria	Kocuria rosea strain DSM 20447	99.78070175	1365/1368

Table S2. ^{1}H and ^{13}C NMR (250 and 63 MHz, respectively; $CDCl_3$) data of **1-6**

Position	**1**		**2**		**3**		**4**		**5**		**6**	
	δ_C	δ_H (m, *J* in Hz)	δ_C	δ_H (m, *J* in Hz)	δ_C	δ_H (m, *J* in Hz)	δ_C	δ_H (m, *J* in Hz)	δ_C	δ_H (m, *J* in Hz)	δ_C	δ_H (m, *J* in Hz)
2	154.9		160.5		158.6		159.1		159.6		161.4	
3	105.0	8.13 (s)	102.8	8.01 (s)	105.8	8.19 (s)	99.9	8.09 (s)	104.5	8.06 (s)	106.7	8.31 (s)
4	168.4		167.3		167.2		160.6		166.0		164.8	
4-OCH_3	56.9	4.16 (s)	56.5	4.12 (s)	56.8	4.14 (s)	56.4	4.19 (s)	56.7	4.11 (s)	57.2	4.17 (s)
5	121.1		117.7		127.4		136.3		127.1		131.9	
5-SCH_3	18.5	2.42 (s)	17.6	2.36 (s)	18.1	2.54 (s)						
5-$SOCH_3$									39.0	3.04 (s)	40.1	3.11 (s)
6	152.7		155.0		137.4		154.3		151.1		132.9	
7	140.4	8.70 (d, 0.5)	62.6	4.93 (s)	116.6		156.4	9.12 (s)	147.2	8.76 (s)	114.7	
2'	153.2		155.6		153.9		155.7		154.4		153.0	
3'	121.2	8.12 (ddd, 8.0, 1.0, 1.0)	121.3	8.44 (ddd, 8.0, 1.0, 1.0)	121.9	8.47 (ddd, 7.6, 4.8, 1.1)	121.7	8.55 (ddd, 8.0, 1.0, 1.0)	122.3	8.47 (d, 7.9)	122.4	8.53 (ddd, 7.9, 1.0, 0.9)
4'	137.7	7.87 (ddd, 8.0, 7.6, 1.7)	137.2	7.84 (ddd, 8.0, 7.6, 1.8)	137.4	7.85 (ddd, 7.9, 7.6, 1.8)	137.3	7.87 (ddd, 8.0, 7.6, 1.8)	137.5	7.84 (ddd, 7.9, 7.6, 1.4)	137.6	7.89 (ddd, 7.9, 7.6, 1.8)
5'	125.1	7.40 (ddd, 7.6, 4.8, 1.0)	124.4	7.34 (ddd, 7.6, 4.8, 1.0)	125.0	7.38 (ddd, 7.9, 1.1, 0.9)	124.5	7.38 (ddd, 7.6, 4.8, 1.0)	125.0	7.35 (dd, 7.6, 4.8)	125.7	7.43 (ddd, 7.6, 4.8, 1.0)
6'	149.6	8.71 (ddd, 4.8, 1.7, 1.0)	149.3	8.68 (ddd, 4.8, 1.8, 1.0)	149.2	8.66 (ddd, 4.8, 1.8, 0.9)	149.2	8.70 (ddd, 4.8, 1.8, 1.0)	149.1	8.65 (d, 4.8)	149.4	8.69 (ddd, 4.8, 1.8, 0.9)
OH				4.78 (brs)						11.2 (brs)		

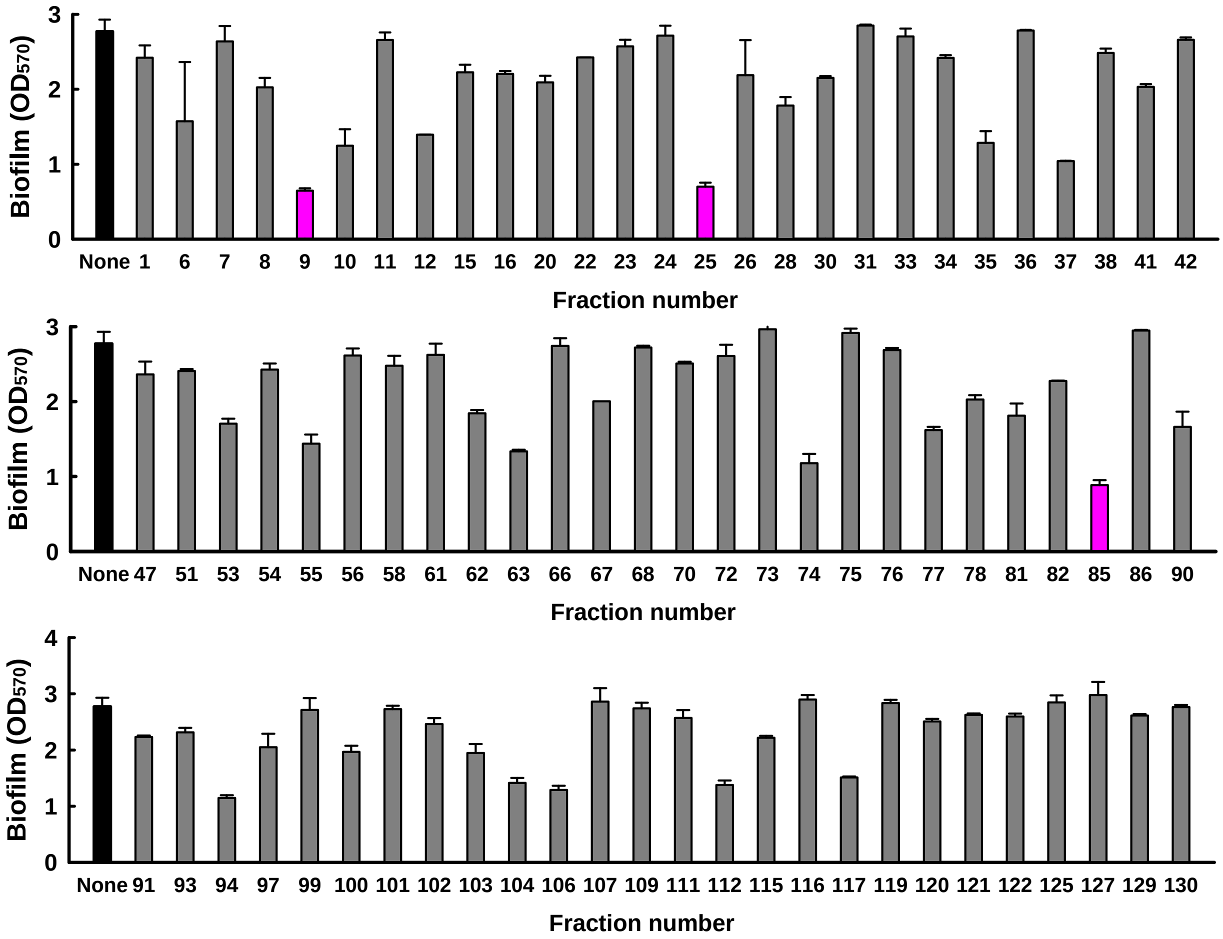

Figure S1. Antibiofilm activity screening of the extracts of 79 bacterial culture broth

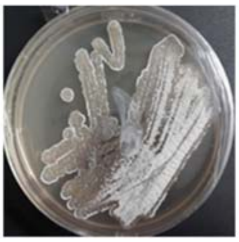

Figure S2. Plate image of *Streptomyces* sp. MC025

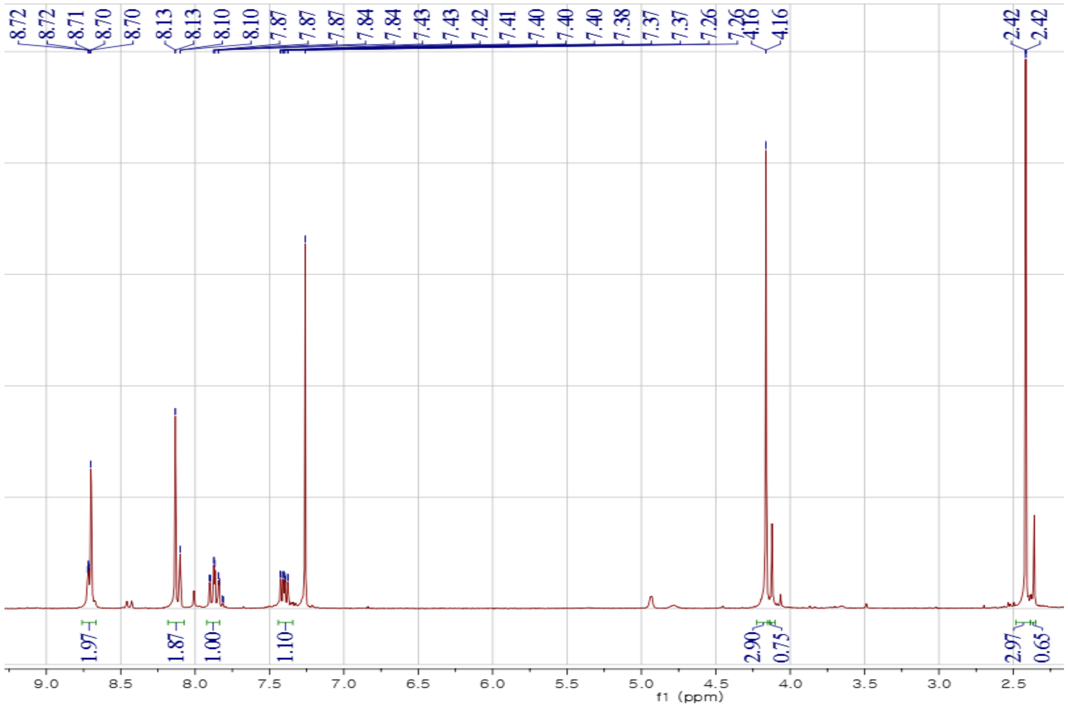

Figure S3. ^{1}H NMR spectrum of compound **1** ($CDCl_3$, 250 MHz)

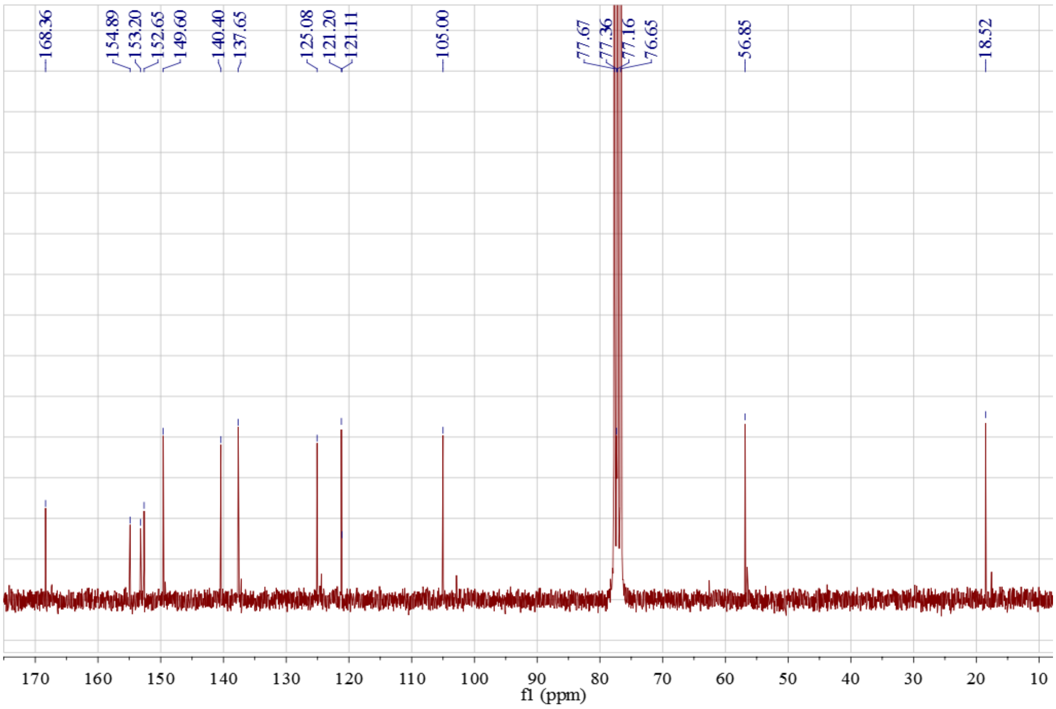

Figure S4. ^{13}C NMR spectrum of compound **1** ($CDCl_3$, 63 MHz)

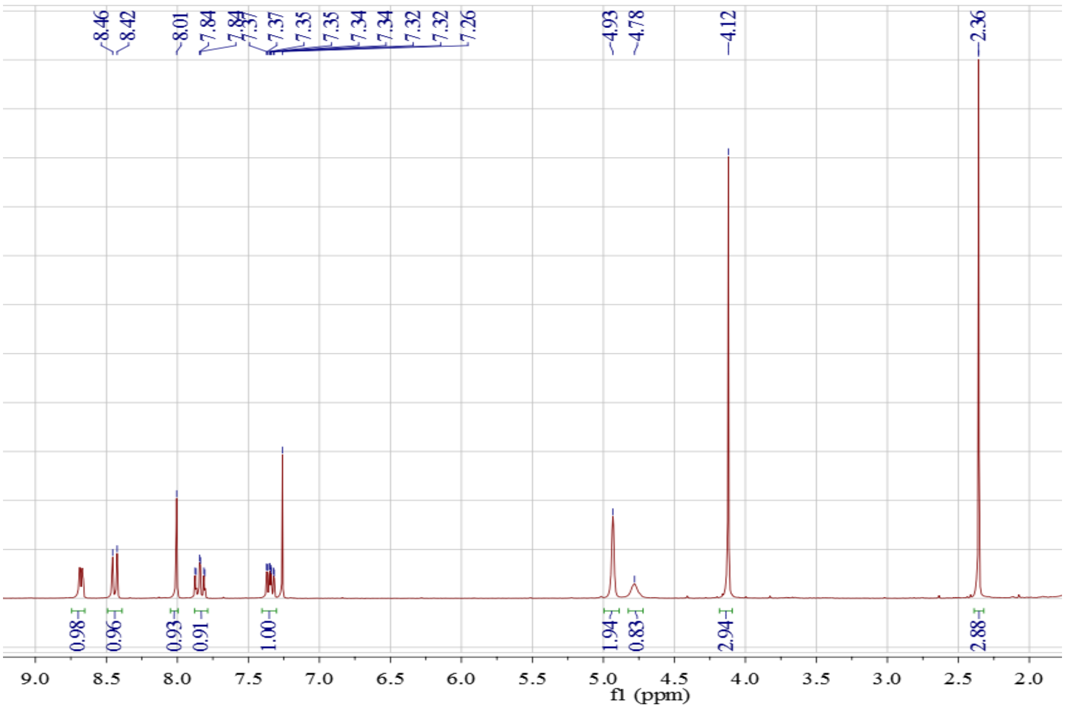

Figure S5. ^{1}H NMR spectrum of compound **2** ($CDCl_3$, 250 MHz)

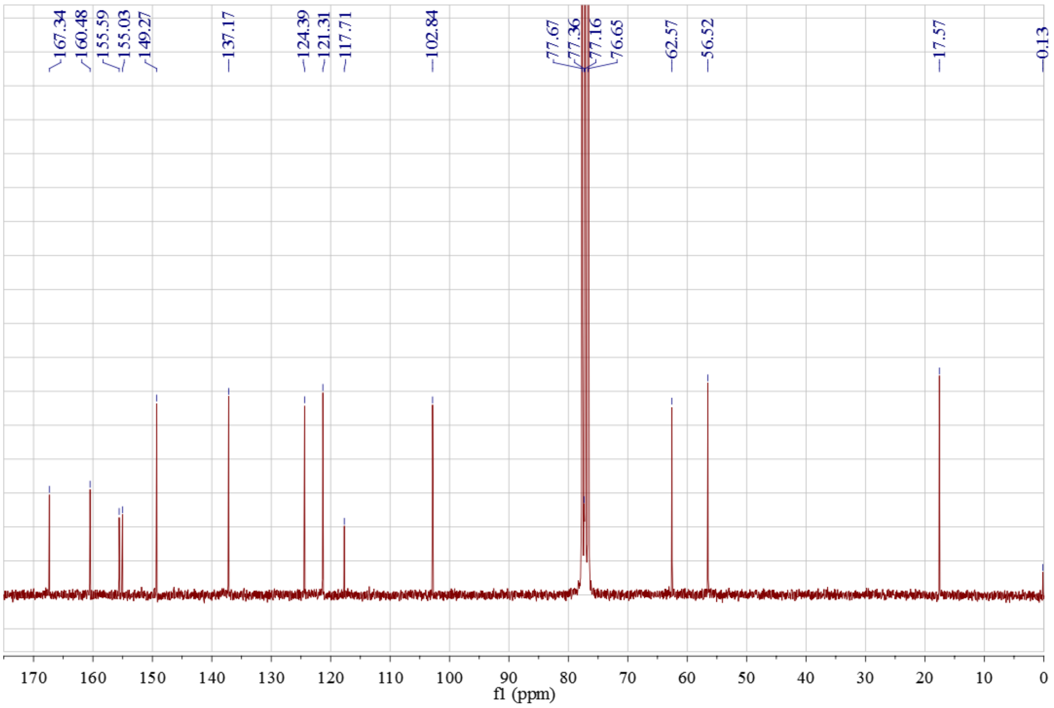

Figure S6. ^{13}C NMR spectrum of compound **2** ($CDCl_3$, 63 MHz)

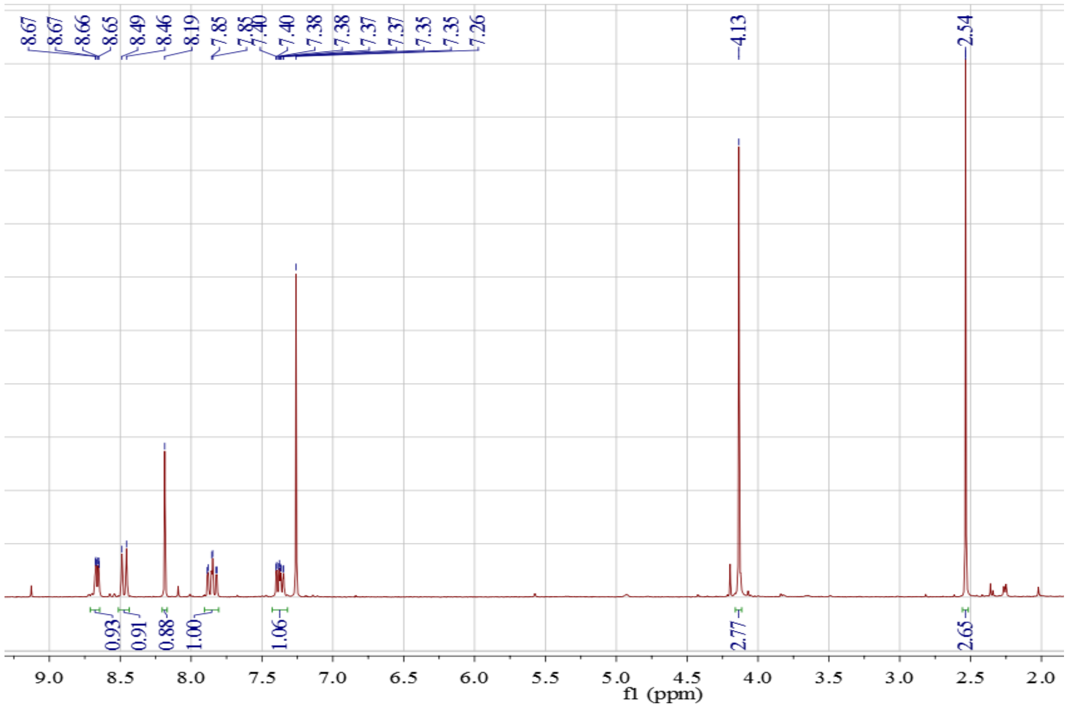

Figure S7. ^{1}H NMR spectrum of compound **3** ($CDCl_3$, 250 MHz)

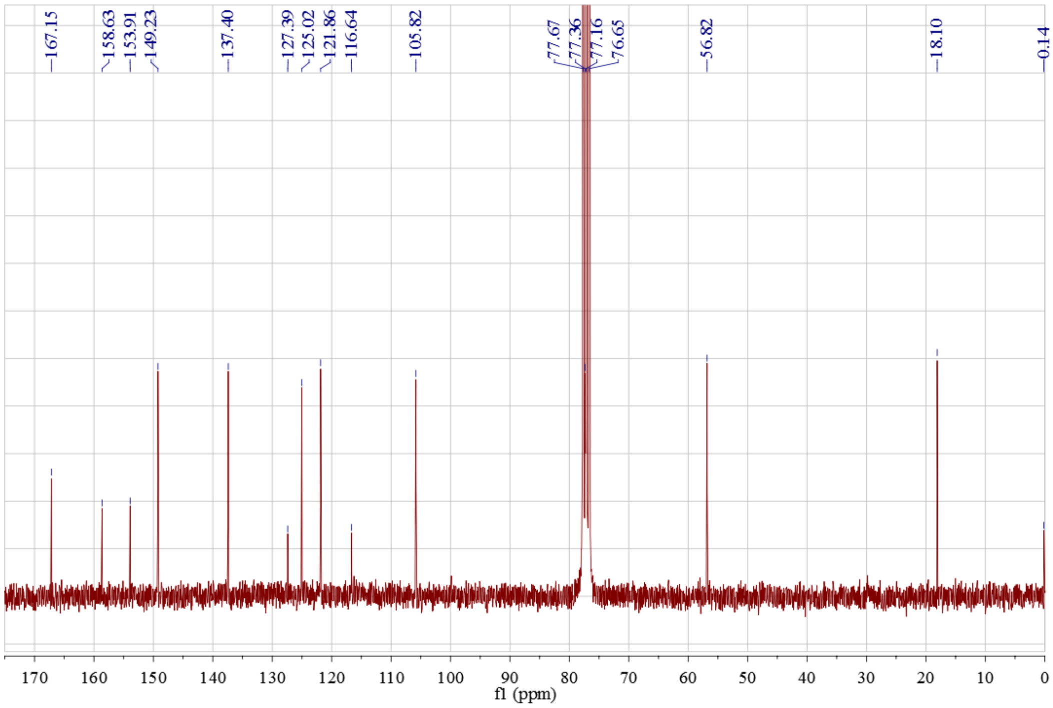

Figure S8. ^{13}C NMR spectrum of compound **3** ($CDCl_3$, 63 MHz)

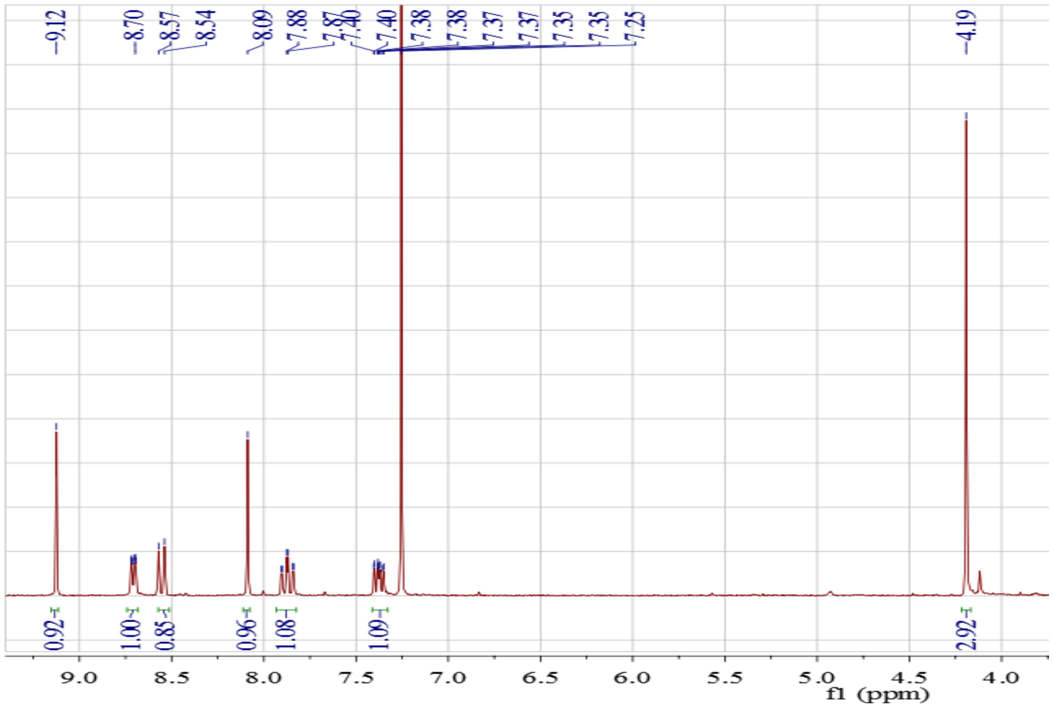

Figure S9. ^{1}H NMR spectrum of compound **4** ($CDCl_3$, 250 MHz)

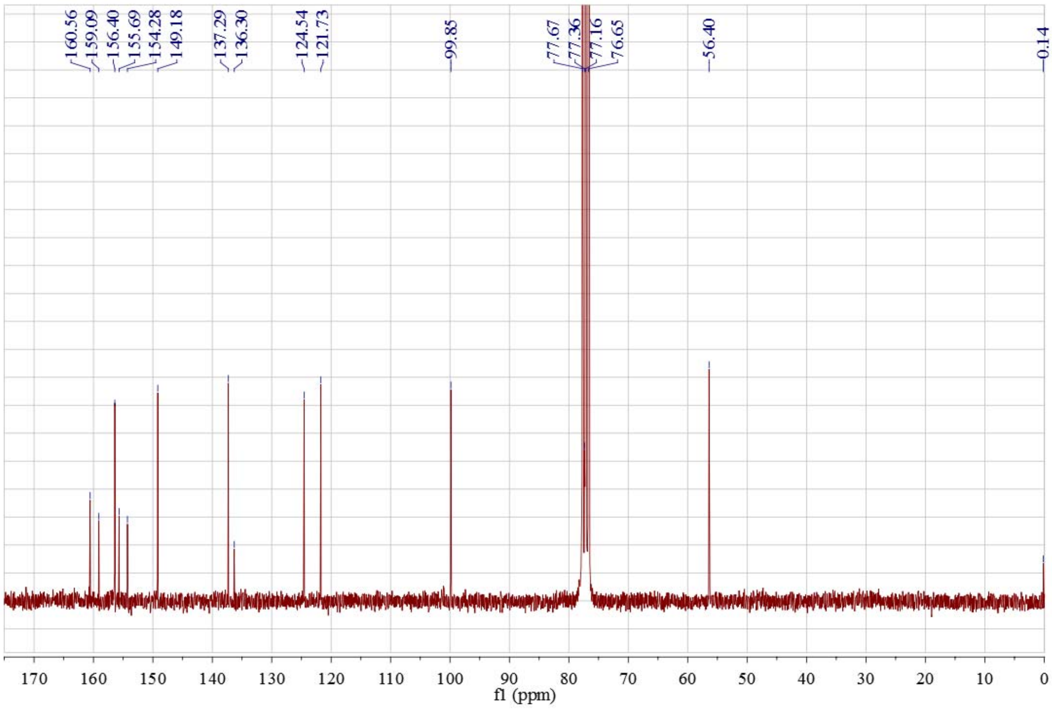

Figure S10. ^{13}C NMR spectrum of compound **4** ($CDCl_3$, 63 MHz)

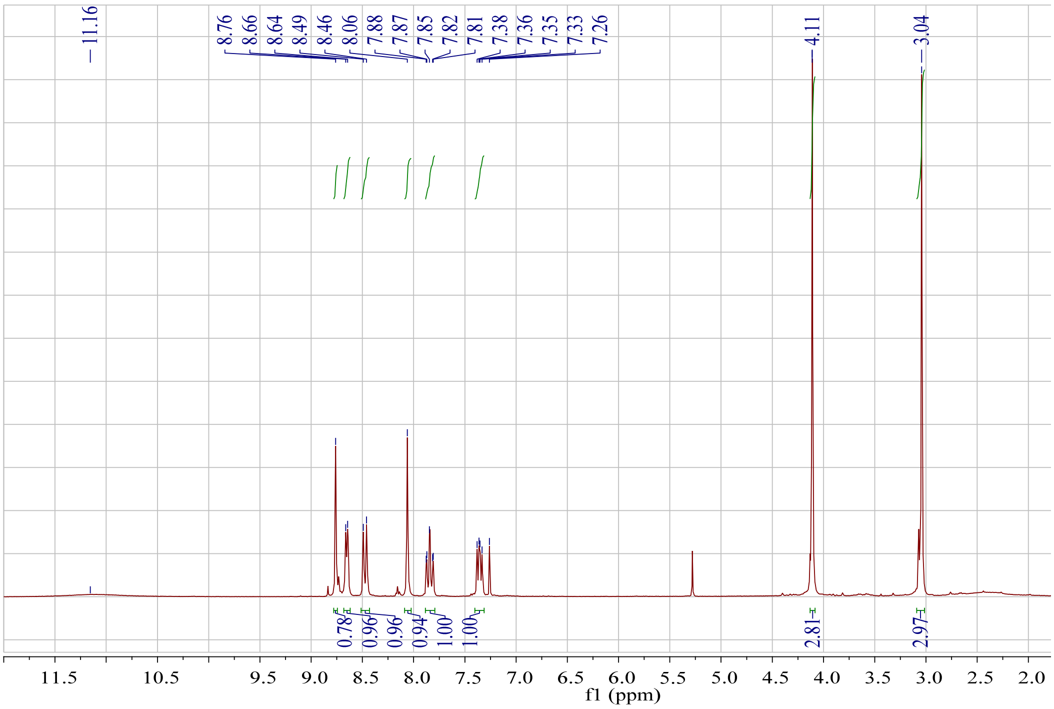

Figure S11. ^{1}H NMR spectrum of compound **5** ($CDCl_3$, 250 MHz)

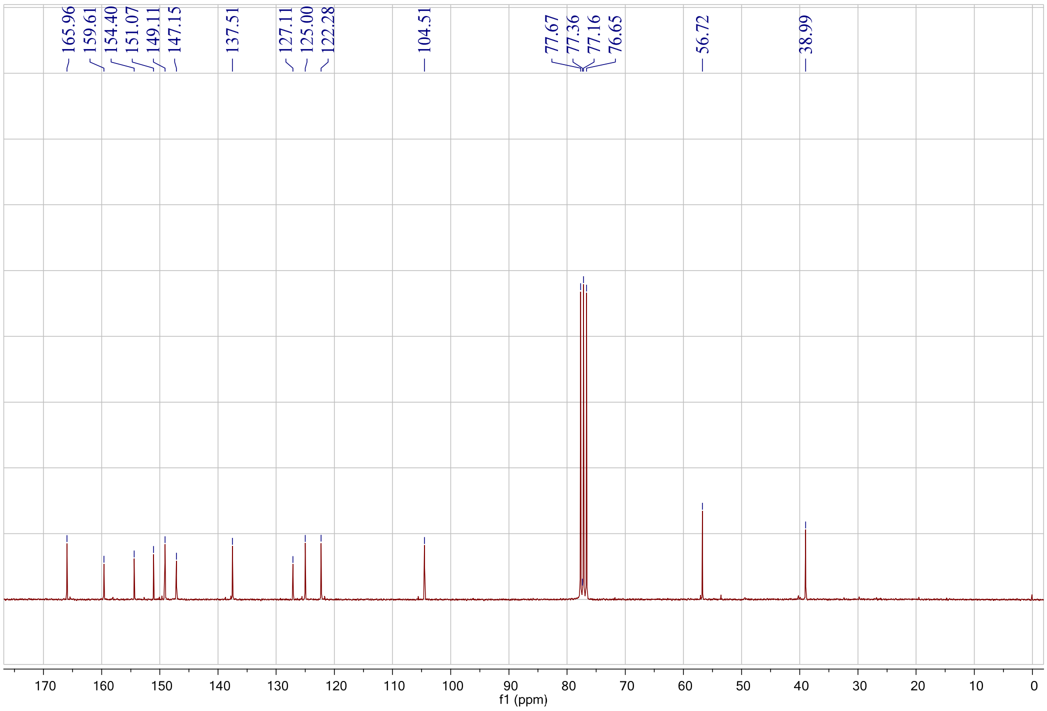

Figure S12. ^{13}C NMR spectrum of compound **5** ($CDCl_3$, 63 MHz)

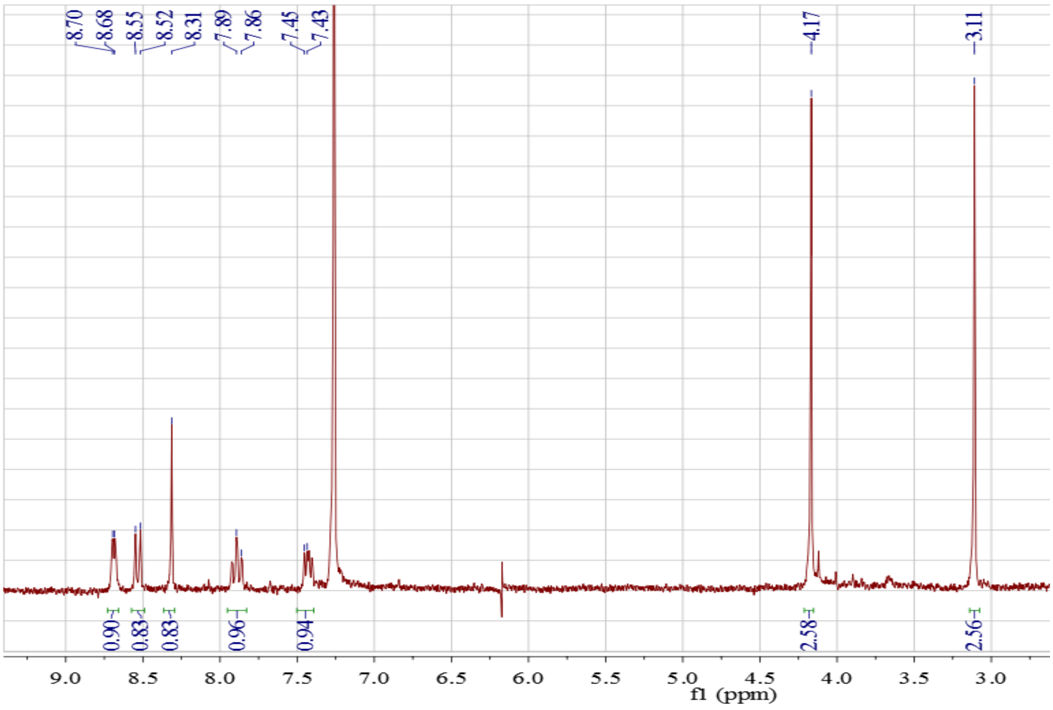

Figure S13. ^{1}H NMR spectrum of compound **6** ($CDCl_3$, 250 MHz)

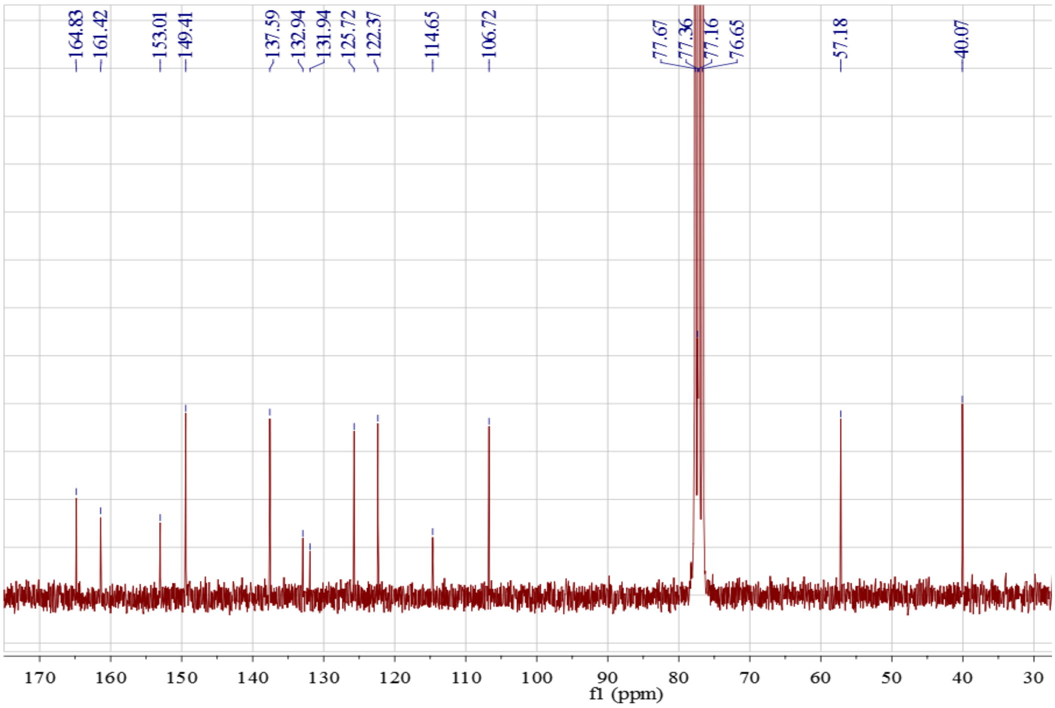

Figure S14. ^{13}C NMR spectrum of compound **6** ($CDCl_3$, 63 MHz)
